# Post COVID-19 Condition in Children and Adolescents: An Emerging Problem

**DOI:** 10.3389/fped.2022.894204

**Published:** 2022-05-11

**Authors:** Jon Izquierdo-Pujol, Sara Moron-Lopez, Judith Dalmau, Alba Gonzalez-Aumatell, Clara Carreras-Abad, Maria Mendez, Carlos Rodrigo, Javier Martinez-Picado

**Affiliations:** ^1^IrsiCaixa AIDS Research Institute, Badalona, Spain; ^2^CIBER de Enfermedades Infecciosas, Madrid, Spain; ^3^Department of Pediatrics, Germans Trias i Pujol University Hospital, Autonomous University of Barcelona, Badalona, Spain; ^4^Department of Infectious Disease and Immunity, University of Vic-Central University of Catalonia (UVic-UCC), Vic, Spain; ^5^Catalan Institution for Research and Advanced Studies (ICREA), Barcelona, Spain

**Keywords:** COVID-19, post COVID-19 condition, long-COVID-19, long-haul COVID, post-acute COVID-19 syndrome, children, adolescents

## Abstract

The severe acute respiratory syndrome coronavirus 2 (SARS-CoV-2) infection became a pandemic in 2020 and by March 2022 had caused more than 479 million infections and 6 million deaths worldwide. Several acute and long-term symptoms have been reported in infected adults, but it remains unclear whether children/adolescents also experience persistent sequelae. Hence, we conducted a review of symptoms and pathophysiology associated with post-coronavirus disease 2019 (post-COVID-19) condition in children and adolescents. We reviewed the scientific literature for reports on persistent COVID-19 symptoms after SARS-CoV-2 infection in both children/adolescents and adults from 1 January 2020 to 31 March 2022 (based on their originality and relevance to the broad scope of this review, 26 reports were included, 8 focused on adults and 18 on children/adolescents). Persistent sequelae of COVID-19 are less common in children/adolescents than in adults, possibly owing to a lower frequency of SARS-CoV-2 infection and to the lower impact of the infection itself in this age group. However, cumulative evidence has shown prolonged COVID-19 to be a clinical entity, with few pathophysiological associations at present. The most common post-COVID-19 symptoms in children/adolescents are fatigue, lack of concentration, and muscle pain. In addition, we found evidence of pathophysiology associated with fatigue and/or headache, persistent loss of smell and cough, and neurological and/or cardiovascular symptoms. This review highlights the importance of unraveling why SARS-CoV-2 infection may cause post-COVID-19 condition and how persistent symptoms might affect the physical, social, and psychological well-being of young people in the future.

## Introduction

Severe acute respiratory syndrome coronavirus 2 (SARS-CoV-2) is the pathogen responsible for coronavirus disease 2019 (COVID-19). Infection by this virus was declared a pandemic by the World Health Organization (WHO) in March 2020 (1). During the SARS-CoV-2 pandemic, the significant efforts made to define the main factors leading to serious illness seemed to focus on the presence of comorbidities, especially advanced age (2). Data provided by the Centers for Disease Control and Prevention (CDC) (March 2022) revealed a total of more than 970,000 deaths, with a COVID-19 mortality rate of 1.2% in the United States of America. Mortality in children is lower than in adults, and it has been widely reported that the disease is less severe in this population (3–5) of children, with mostly mild and asymptomatic cases. Although the vast majority of children and adults with COVID-19 do not experience any change and live normally after the acute infection, it has recently been reported that some people (both adults and children) cannot recover their previous health after being infected by SARS-CoV-2, as they have long-term symptoms, such as persistent cough, fatigue, altered taste and/or smell, and memory loss. The scientific community refers to this group of persistent symptoms as “long-COVID-19,” “post-acute COVID-19 syndrome” (PACS), or “post-COVID-19 condition,” which is the preferred term of the WHO (6). While some researchers are trying to unravel the mechanisms causing persistent symptoms in adults, few studies focus on children with post-COVID-19 condition. In this study, we aimed to review and summarize current knowledge on post-COVID-19 condition in children, with emphasis on clinical symptoms and potential pathophysiologic mechanisms, and to share our experience in the Pediatric Persistent COVID Unit of the Germans Trias Hospital (Badalona, Spain).

## From Acute Disease to Post-COVID-19 Condition

**FIGURE 1:**
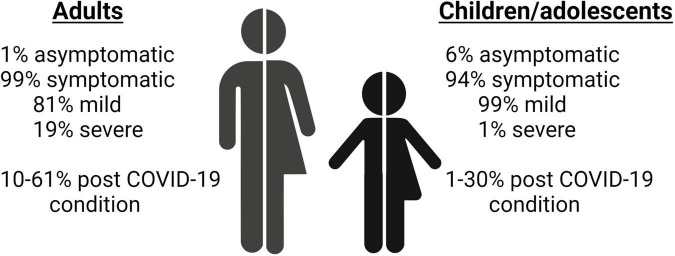
Diagram comparing the frequencies of acute and post-COVID-19 condition between adults and children/adolescents (3–5, 31, 32, 35–38, 42–44).

The clinical features and symptoms of acute COVID-19 have been widely described and reviewed (7–11). The most common symptoms in adults include fever, fatigue, and dry cough. Although infected children experience similar symptoms, the severity of acute infection in this group is lower than in adults. Several reports (3–5) show that 5.9% of children are asymptomatic (compared with 1% of adults) and that the infection is mild in 99.3% of cases in children (compared with 81% in adults) (Figure 1). A study performed in China with 341 children aged 4–14 years (median, 7 years) found that the most common symptoms were fever (77.9%), cough (32.4%), and diarrhea (4.4%) (3). Of note, multisystem inflammatory syndrome (MIS), in which various body parts can become inflamed (heart, lungs, kidneys, brain, skin, eyes, and gastrointestinal organs), has been associated with COVID-19. Despite data showing that acute infection is not as severe in children as in adults, a recent study suggested that MIS in children (MIS-C) is more common than previously thought (12). The authors analyzed the symptoms of 1,200 hospitalized children with a median age of 4.7 years and observed that 10.6% had MIS-C during the infection (127/1,200). In this same study, 3 patients with MIS-C eventually died, although they had serious comorbidities, such as acute leukemia and bone marrow transplant, overweight, and malignant neoplasm. MIS-C due to COVID-19 has been analyzed elsewhere (13). MIS affecting adults is less common, although it has been demonstrated and described in patients with severe COVID-19 (14–16).

Vertical transmission of SARS-CoV-2 has been reported in pregnant women (17, 18), and while it seems to be rare (3.2% in a review of 38 studies (18)), a recently published article (19) demonstrated that SARS-CoV-2 can infect trophoblasts in the placenta and provoke fetal demise in 2.5% of cases (5 out of 198 placental tissue samples).

One interesting trait during the pandemic was that some children developed chilblains (more common than in adults). In an earlier investigation (20), it was concluded that chilblains were not associated with COVID-19, as none of the patients were positive for SARS-CoV-2 antigens, nucleic acids, or SARS-CoV-2 antibodies. However, this was later contradicted in a recently published review (21), where the authors found an association between the spike protein and chilblains and reported that type 1 interferon (IFN) production was important for the development of chilblains, even if patients remained asymptomatic or had negative antibody titers.

Although symptoms resolve satisfactorily in most acutely infected adults, a significant percentage of patients with COVID-19 experience long-lasting symptoms weeks or months after infection. Long-term symptoms after severe viral infection are not novel, as post-viral syndrome and sequalae after viral infections have previously been described (22–27). Moreover, similar symptoms, such as fatigue or persistent shortness of breath, were previously described by survivors of SARS and Middle East respiratory syndrome (MERS) (28–30). The novelty of post-COVID-19 condition is that it is independently associated with the severity of the acute illness, according to a prospective cohort study of 312 adult patients (247 self-isolating patients and 65 hospitalized) (31). In this study, 61% of patients had persistent symptoms 6 months after the acute phase of the infection, regardless of symptoms and disease severity. The most common symptoms in self-isolating patients with COVID-19 after 6 months included fatigue (30%), absence of or disturbed taste or smell (27%), concentration problems (19%), memory problems (18%), and dyspnea (15%). Similar symptoms were found in another study based on an online survey of 3,762 participants (32), with fatigue being the dominant symptom 6 months after infection. Interestingly, 45.2% of those surveyed required shorter work hours than before their illness, likely owing to the long-lasting effect of their COVID-19 symptoms. A recent report (33) also highlighted fatigue, dyspnea, cough, headache, loss of taste and/or smell, and cognitive or mental health impairment as the most common symptoms in people with post-acute sequelae of SARS-CoV-2 infection. Moreover, age > 40 years, female sex, reverse transcription PCR (RT-PCR) low cycle threshold (Ct) values, and ageusia have been reported to be associated with persistent symptoms after COVID-19 in adults (34).

Recent studies have shown that children also experience post COVID-19 condition. In a preliminary report of 5 cases (35), all children reported fatigue and dyspnea 2 months after acute infection. However, no specific PCR-based diagnosis of SARS-CoV-2 was available, and 4 did not develop specific antibodies. A second study conducted in Italy with 129 children diagnosed with SARS-CoV-2 infection also showed evidence of post-COVID-19 condition (36). The most persistent symptoms were insomnia (18.6%), respiratory symptoms (such as pain and chest tightness) (14.7%), nasal congestion (12.4%), fatigue (10.8%), concentration difficulties (10.1%), and muscle pain (10.1%). The study also showed that 42.6% of patients presented at least 1 symptom 60 days after acute infection. Similar findings were reported in a study of 58 children and adolescents (37), where 44.8% reported symptoms of post-COVID-19 condition, the most common being fatigue (21%), shortness of breath (12%), exercise intolerance (12%), weakness (10%), and walking intolerance (9%). Another report (38) (the CLoCk study) found the most common persistent symptoms in children and adolescents who tested positive for SARS-CoV-2 to be sore throat, headache, tiredness, and loss of smell. Similar results were reported in a Dutch study (39), where the most common complaints were fatigue, dyspnea, and concentration difficulties in 89 children suspected of having post-COVID-19 condition. A recently published pre-print meta-analysis, reviewing 68 articles focused on post-COVID-19 condition in children and adolescents, showed that the most prevalent clinical manifestations were mood symptoms (16.5%), fatigue (9.7%), and sleep disorders (8.4%); and more interestingly that, when compared with controls, children infected by SARS-CoV-2 had a higher risk of persistent dyspnea (odds ratio [*OR*] 2.69 95% *CI* 2.30–3.14), anosmia/ageusia (*OR* 10.68, 95% *CI* 2.48, 46.03), and/or fever (*OR* 2.23, 95% CI 1.22–4.07) (40).

In children, an interesting comparison was recently reported between persistent symptoms after COVID-19 and those related to non–SARS-CoV-2 infection (41). Persistent symptoms of 236 pediatric patients with COVID-19 were compared with those of 142 children with non–SARS-CoV-2 viruses. It was concluded that persistent symptoms were more apparent in COVID-19 than in any other SARS-CoV-2 infection, thus highlighting the importance of post-COVID-19 condition in children.

## Frequency of Post-COVID-19 Condition in Children and Adolescents

As observed in the study by Buonsenso et al. conducted in Italy (36), a high percentage of children had at least 1 symptom 60 days after acute infection, suggesting that post-COVID-19 condition in children is a major problem that may have been underestimated. More recent data and reports from the United Kingdom’s Office for National Statistics (ONS) (42) showed varying results on the prevalence of post-COVID-19 condition in adults and children with confirmed SARS-CoV-2 infection. Data reported up to 1 April 2021 showed that 9.8% of infected children aged 2–12 years and 13% of those aged 12–16 years reported long-lasting symptoms at least 5 weeks after infection. These rates increased with age, peaking in the 35–49-year age group, where 25.6% of infected people reported long-lasting symptoms at least 5 weeks after infection. After 12 weeks of acute infection, the frequency of persistent symptoms decreased to 7.4% in children aged 2–12 years and to 8.2% in adolescents aged 12–16 years. A recently published study, which analyzed 1,734 children in the United Kingdom who tested positive for SARS-CoV-2 (43), revealed lower rates than previously seen in the ONS data, where 4.4% of children had long-lasting symptoms at least 28 days after acute infection, i.e., a lower frequency than in the previous study. Similar findings were reported in a study that analyzed data from 4,678 children in England and Wales (44), where only 174 had a history of SARS-CoV-2 infection (4.6% with persistent symptoms). In contrast, a recently published pre-print meta-analysis showed a prevalence of 25.2% (40). Despite the variability in persistence between studies, post-COVID-19 condition is clearly a more important health condition than initially thought.

## Acute vs. Post-COVID-19 Condition Symptoms in Children and Adolescents

**FIGURE 2:**
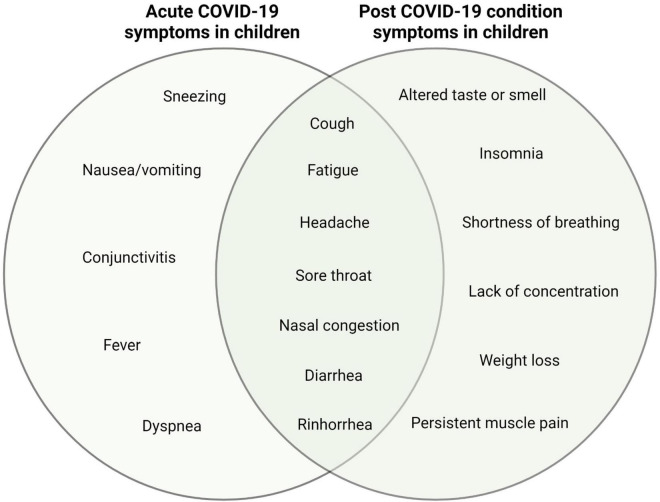
Diagram representing specific and shared symptoms of acute and post-COVID-19 condition in children and adolescents.

It is very important to identify the differential symptoms of post-COVID-19 condition in children and adolescents to facilitate diagnosis. However, although some symptoms are exclusively related to post-COVID-19 condition, the most common symptoms are shared with acute infection. Fatigue is the most frequent symptom of post-COVID-19 condition in children and adolescents, with percentages varying between 10.8 and 20.1% depending on the study. Fatigue is also one of the symptoms most frequently associated with the acute phase of the disease, and percentages vary from 2.2 to 9% depending on the study. Headache, which is present in acute COVID-19, is also a highly prevalent symptom during post-COVID-19 condition. Other common symptoms during post-COVID-19 condition include cough and diarrhea (more prevalent during acute illness), sore throat, nasal congestion, and rhinorrhea. Symptoms exclusively associated with the post-COVID-19 condition in children differ between studies but include taste and/or smell disturbances, insomnia, shortness of breath, poor concentration, weight loss, and persistent muscle pain. On the other hand, the main symptoms associated exclusively with acute disease are fever, sneezing, nausea/vomiting, conjunctivitis, and dyspnea (Figure 2).

## Pathophysiology of Post-COVID-19 Condition: An Area That Has Yet to Be Unraveled

**FIGURE 3:**
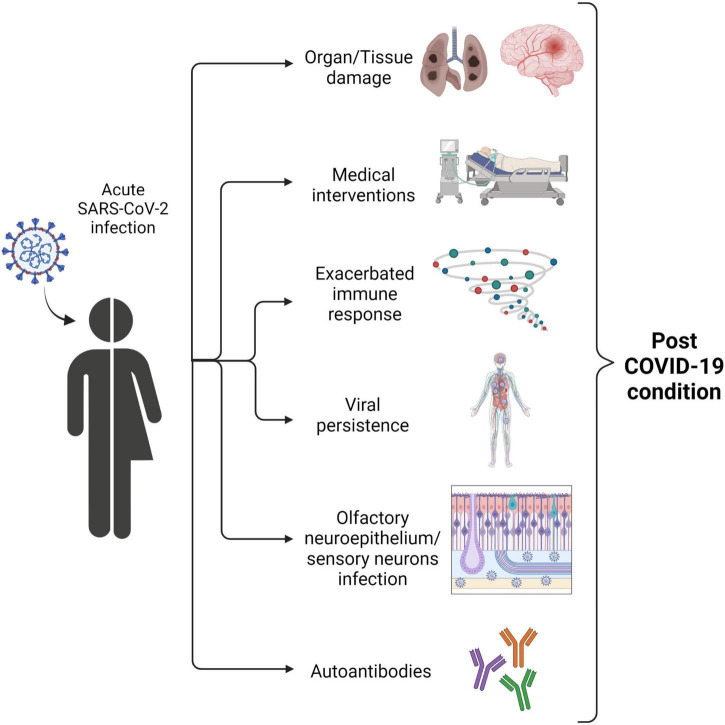
Diagram showing potential pathophysiological mechanisms of post-COVID-19 condition symptoms.

Although there is clear evidence that post-COVID-19 condition is pathological in both children and adults, the pathophysiology of this disease remains unknown. However, several hypotheses have been put forward (Figure 3). The predictors of post-COVID-19 condition in children and adolescents include older age, muscle pain at hospital admission, and admission to the intensive care unit (ICU) during acute infection (37). As occurs in adults, the risk associated with initially severe COVID-19 has been associated with the exacerbated immune response that can lead to organ damage, as well as the medical and therapeutic interventions required, which may cause lasting sequelae. Moreover, the virus has been shown to persist in intestinal biopsies at 4 months after the onset of COVID-19 (45), suggesting that viral persistence might/could be associated with post-COVID-19 condition.

A recent study comparing the immune profiles of children who recovered fully from COVID-19 and those with long-term symptoms (46) revealed persistently high levels of interleukin (IL)-6 and IL-1β, thus attributing a relevant role to the innate immune response during the post-COVID-19 condition. Since IL-6 and IL-1β act as mediators in the inflammatory response, persisting levels of these cytokines could explain persistent symptoms, such as fatigue or headache after acute illness.

As mentioned above, loss of taste and/or smell is a common symptom during persistent COVID-19. SARS-CoV-2 uses angiotensin-converting enzyme 2 (ACE2) as the entry receptor to infect cells. ACE2 is expressed in cells of the human respiratory system (47, 48). Although SARS-CoV-2 predominantly affects the respiratory tract, studies in human brain organoids have shown that SARS-CoV-2 can also infect neurons (49, 50). Moreover, a recent report (51) found evidence that the olfactory neurepithelium (olfactory sensory neurons, support cells, and immune cells) can be infected by SARS-CoV-2. The study was carried out with samples from seven patients with COVID-19 who presented with acute loss of smell. The authors showed the presence of viral transcripts and SARS-CoV-2–infected cells in olfactory mucosa samples from patients with long-term persistent anosmia due to COVID-19, suggesting that the loss of smell in patients with symptoms persisting after several months of infection could be due to persistence of SARS-CoV-2 and inflammation of the olfactory neurepithelium.

Persistent cough is one of the most common symptoms of the post-COVID-19 condition. The potential underlying mechanism was discussed in a recent personal view published in April 2021 (52). The authors suggested that persistent cough might be associated with neuroinflammatory and neuroimmune mechanisms related to vagal sensory nerves. These mechanisms resemble those that infect the olfactory neurepithelium, highlighting that some symptoms reported in post-COVID-19 condition could be related to the potential neurotropism of SARS-CoV-2.

Autoimmune diseases triggered by infection are well documented and include rheumatic fever and Guillain-Barré syndrome (53, 54). Several reports have already demonstrated that autoantibodies are generated during COVID-19 according to the severity of the disease (55–57). Moreover, a recent study revealed the presence of autoantibodies against G-protein coupled receptors (GPCRs) in patients with post-COVID-19 condition (58). Given that GPCRs can disrupt the balance of neuronal and vascular processes, their presence could explain some of the neurological and/or cardiovascular symptoms observed in post-COVID-19 condition.

## Can Genetics Predispose to Post-COVID-19 Condition?

As of March 2022, the role of genetics in post-COVID-19 condition remains unclear. Most studies investigate genetic factors that might explain differences in the course of acute SARS-CoV-2 infection, including those related to innate errors of immunity (59). Moreover, several studies had reported several genetic factors associated with severe COVID-19 (60–63), such as epigenomic markers (61), blood group (60), and traits associated with protection against severe disease (63).

A search of clinicaltrials.gov based on the terms “host” & “genetics” & “COVID” revealed 40 studies attempting to unravel the link between the genotype and susceptibility to infection. However, when using the terms “host” & “genetics” & “long-COVID” or “post COVID-19 condition” or “post-acute COVID syndrome,” we found no studies exploring the potential contribution of genetic factors to post-COVID-19 condition. Therefore, it could be interesting to design studies aimed at identifying potential genotypic markers of predisposition to post-COVID-19 condition in both adults and children.

## Sociopsychological Causes of Post-COVID-19 Condition: SARS-CoV-2 Infection or Lockdown?

In addition to the pathological effects previously described in pediatric post-COVID-19 condition, other sociopsychological conditions related to lockdown may have contributed to the symptoms of post-COVID-19 condition. A study conducted in 1,560 students in the United Kingdom (median age, 15 years, interquartile [IQR] [14–17]) (64) tried to determine whether there were differences in symptoms between seropositive children and seronegative children. The study found no significant differences between the symptoms of the 1,356 SARS-CoV-2–seronegative children and the 188 seropositive children, suggesting that most of the symptoms could be due to lockdown syndrome rather than viral infection. However, this study is limited to a very specific age range, suggesting that there is a need for alternative studies covering several pediatric age ranges.

A more recent report (38) showed that having 3 or more persistent symptoms at 3 months after testing was more common in PCR-positive children (30.3%) than in PCR-negative children (16.2%). The conclusions of the report are similar to those of a recent systematic review of 23 studies on persistent COVID-19 symptoms in children (65). Both highlight the importance of having a SARS-CoV-2–negative control group to assess the real differences between persistent symptoms.

In addition, a report by P. Zimmermann and colleagues noted the challenges of studying post-COVID-19 condition (66). After the examination of 27 studies, they found a large variation in results, highlighting how difficult it is to study post-COVID-19 condition. Therefore, better guidelines, characterization of exclusive post-COVID-19 condition symptoms and studies with an uninfected control group must be drawn up to distinguish between long-term symptoms caused by SARS-CoV-2 infection and pandemic-related symptoms (67).

## Our Experience in the Pediatric Persistent Covid Unit of the Germans Trias Hospital

In December 2020, Germans Trias i Pujol University Hospital (Barcelona, Spain) created one of the first Pediatric Persistent COVID Units in Spain and is currently following a cohort of 120 children/adolescents with post-COVID-19 condition from the metropolitan area of Barcelona. The unit consists of a multidisciplinary team, such as pediatricians and other medical specialists (infectious disease specialists, pulmonologists, neurologists, cardiologists, and nutritionists), and experts in rehabilitation and physical therapy, psychiatry, psychology, radiology, and neuropsychology who participate in the clinical study and treatment of post-COVID-19 condition in children and adolescents. The unit has also partnered with research groups specialized in virology, immunology, and genetics to perform an in-depth analysis of the causes of and the mechanisms underlying this phenotype.

The cohort includes pediatric patients younger than 18 years old, diagnosed with SARS-CoV-2 infection and with at least 12 weeks of persistent COVID-19 symptoms after COVID-19 disease. The median age of the cohort is 14 years (IQR, 12.2–15.8; 66% female), and patients have at least 3 symptoms associated with post-COVID-19 condition (Table 1). The most common symptoms are asthenia/fatigue (98%), headache (75%), muscle weakness (74%), dyspnea (68%), myalgia/arthralgia/paresthesia (64%), and cognitive neurological disorders (decreased attention) (44%). These had been present for more than 6 months in 36% of patients.

**TABLE 1:** Demographic and clinical characteristics of the pediatric post–coronavirus disease 2019 (COVID-19) condition study cohort (pediaCOVID, *n* = 50) at the Germans Trias i Pujol Hospital.

Characteristics	Value
Age, median (IQR)	14.1 (12.2–15.8)
Female sex, n (%)	33 (66)
Number of acute symptoms, median (IQR)	6 (4–8)
Days of duration of acute symptoms, median (IQR)	10 (4.8–20.3)
Number of post COVID-19 condition symptoms, median (IQR)*	10 (7–16)

*List of considered post-COVID-19 condition symptoms: asthenia/fatigue, muscular weakness, neurocognitive neuro disorders, headaches, dyspnea, myalgia/arthralgias, insomnia, chest oppression/pains, orthostatic hypotension, hyporexia/anorexia, deafness/tinnitus/sonophobia, anosmia, ageusia/dysgeusia, abdominal pains, palpitations/tachycardia, paresthesia, photophobia, dizziness/vertigo, cough, skin signs, diarrhea, weight loss, epigastric pains/dyspepsia/food impaction, odynophagia/dysphagia, vomiting/nausea, dysphonia, and fever/chills, rhinorrhea.

Fatigue was evaluated using the Pediatric Functional Assessment of Chronic Illness Therapy-Fatigue (pedsFACIT-F) Scale. Most patients (68%) had moderate to very high grades. Health status was evaluated using the Pediatric Quality of Life instrument (PedsQL), which revealed that quality of life was affected in 37.5% of children and adolescents assessed, and psychosocial health in 60%; in particular, 23% had emotional and behavioral problems. Furthermore, because of post-COVID-19 condition, most pediatric patients were unable to attend school full-time (54%) or engage in extracurricular activities (>70%).

These findings further highlight the urgent need for studies to unravel the underlying causes of post-COVID-19 condition and to develop a treatment that will enable patients to return to their pre-COVID-19 health status.

## Conclusion

A recently published viewpoint (68) highlighted the potential legacy of post-COVID-19 condition. There has been significant resistance to recognizing post-COVID-19 condition as a clinical entity in adults and even more so in children. Although clinical guidelines are being developed to facilitate the diagnosis and treatment of post-COVID-19 condition in adults, as well as to study its pathophysiology and sociopsychological causes and consequences, progress in pediatric disease is slower (35–37). To ensure the health of future generations, post-COVID-19 condition should be addressed decisively and effectively, especially in children and adolescents.

## Search Strategy and Selection Criteria

References for this Review were identified through searches of PubMed and Google scholar with the search terms “long-COVID,” “post-acute COVID-19 syndrome,” and “post COVID-19 condition” from January 2020 until March 2022. Articles were also identified through searches of the authors’ own files. Only articles published in English were reviewed. The final reference list was generated on the basis of originality and relevance to the broad scope of this Review.

## Author Contributions

JI-P, SM-L, and JM-P performed the search strategy and selection criteria and wrote and reviewed the manuscript. JD wrote and reviewed the manuscript. AG-A, CC-A, MM, and CR provided the clinical data and reviewed the manuscript. All authors contributed to the article and approved the submitted version.

## Conflict of Interest

The authors declare that the research was conducted in the absence of any commercial or financial relationships that could be construed as a potential conflict of interest.

## Publisher’s Note

All claims expressed in this article are solely those of the authors and do not necessarily represent those of their affiliated organizations, or those of the publisher, the editors and the reviewers. Any product that may be evaluated in this article, or claim that may be made by its manufacturer, is not guaranteed or endorsed by the publisher.
